# Early clinical outcomes of all-inside arthroscopic anterior cruciate ligament reconstruction with autograft tendon augmentation using the LARS internal brace ligament

**DOI:** 10.3389/fbioe.2025.1556106

**Published:** 2025-04-15

**Authors:** Huang Wenlong, Yang Maosheng, Wang Hanbin, Li Yi

**Affiliations:** Department of Joint Surgery and Sports Medicine, Shandong Provincial Hospital Affiliated to Shandong First Medical University, Jinan, China

**Keywords:** anterior cruciate ligament, all-inside reconstruction, internal brace, ligament augmentation and reconstruction system, autograft tendon, mechanical strength, augmentation

## Abstract

**Objective:**

The aim of this study was to compare the early clinical outcomes of all-inside anterior cruciate ligament (ACL) reconstruction using hamstring tendons augmented using the ligament augmentation and reconstruction system (LARS) versus hamstring tendons alone as a control.

**Methods:**

This study included 99 patients with ACL injuries who underwent all-inside arthroscopic ACL reconstruction using either the LARS internal brace ligament combined with hamstring tendon (augmentation group, n = 48) or hamstring tendon alone (hamstring group, n = 51). Postoperative follow-up was conducted using Lysholm, International Knee Documentation Committee (IKDC), Tegner, KOS-ADLS, and ACL-RSI scores to evaluate functional recovery of patients at 1, 3, and 6 months. If necessary, MRI findings obtained at postoperative 3 months were also analyzed to evaluate graft integration and healing dynamics. Tensile strength of the augmented graft was measured through tensile testing. Moreover, to evaluate the postoperative healing status of the augmented tendon, an ACL reconstruction model was established using New Zealand white rabbits. At 4 and 8 weeks postimplantation, rabbit knees were harvested, decalcified, embedded in paraffin, and stained to evaluate new tissue formation. All statistical analyses were conducted using the GraphPad Prism and SPSS software, with appropriate statistical tests applied for comparison between groups.

**Results:**

At 1-month postoperative follow-up, the LARS augmentation group demonstrated significantly higher Lysholm, IKDC, and KOS-ADLS scores than the hamstring group, with P < 0.01 for all comparisons. At 3-month postoperative follow-up, the augmentation group exhibited significantly higher Tegner, Lysholm, IKDC, and KOS-ADLS scores than the hamstring group, with P < 0.05 for all measurements. In the tensile testing, the tendons + LARS and LARS groups showed significantly higher maximum loads and lower elongation than the tendon group with P < 0.001 for maximum load and P < 0.05 for elongation. Examination of the histological sections at 4 and 8 weeks showed that the LARS ligament exhibited excellent biocompatibility, with abundant collagen fibers and neovascularization identified between its fibers.

**Conclusion:**

The combination of LARS internal brace ligaments with autograft tendons in ACL reconstruction provides superior early postoperative outcomes, improving knee stability and patient satisfaction with no remarkable complications. The augmented graft exhibited reliable tensile strength and favorable tissue integration.

## 1 Introduction

The anterior cruciate ligament (ACL) plays a vital role in maintaining knee joint stability during flexion and extension movements. However, due to sports injuries, accidents, or other factors, ACL injuries are becoming increasingly common, accounting for approximately 40% of knee injuries, (Urbanek et al., 2023), with an annual global incidence rate of 30–78 cases per 100,000 individuals (Gans et al., 2018). Moreover, factors such as reduced cell density, insufficient blood supply, and lack of nutrients restrict the regenerative ability of the ACL. Reconstruction of the ACL represents a promising approach for mitigating the challenges posed by its limited regenerative capacity. In this regard, surgical intervention for ACL injuries, when combined with biomechanical reconstruction of the joint, has demonstrated superior clinical outcomes and cost-effectiveness (Beard et al., 2022). ACL reconstruction is recommended for patients aged ≤50 years with symptoms of knee instability, irrespective of their level of physical activity, meniscus status, and OA grade (Cai et al., 2021; Tischer et al., 2023). The primary objective of ACL reconstruction surgery is to reestablish knee joint stability and flexion–extension function during physical activities as well as prevent premature degenerative changes in the joint.

Although various types of grafts are used in ACL reconstruction, including autografts, allografts, and synthetic ligaments, no universally recognized “gold standard” exists for graft choice in ACL reconstruction, because each graft type provides unique benefits and drawbacks (Chen et al., 2024). Although commonly used autografts, such as hamstring tendons and bone-patellar tendon-bone (BPTB) tendons, are equivalent to or superior to the native ACL in terms of mechanical and biomechanical properties (Runer et al., 2023), they are associated with complications such as pain at the donor site and weakness in knee extension (Ajrawat et al., 2021). Additionally, autologous tendon grafts may be relatively thin in patients with muscle atrophy or a lean physique. Using hamstring tendon grafts with a diameter of less than 8 mm significantly increases the risk of ACL reconstruction failure (Paschos, 2022). Although allograft tendons avoid the issues of donor-site complications and insufficient tendon diameter, they carry risks of immune rejection and disease transmission (Cohen and Sekiya, 2007). Moreover, compared to autografts, allografts exhibit higher rates of revision and loosening (Min et al., 2024; Migliorini et al., 2025). Although LARS ligaments have demonstrated sufficient mechanical properties and improved knee function, it is necessary to focus on potential complications such as synovitis, tunnel enlargement, loose fixation, and poor graft bone integration (Tiefenboeck et al., 2015). In addition to graft selection, surgical technique is also crucial. At present, there are two widely recognized surgical techniques: traditional ACL reconstruction and all-inside ACL reconstruction. Compared with the traditional method, all-inside reconstruction has the advantages of bone preservation, shorter graft length requirements, facilitating healing by using autologous tendon graft instead of artificial tendon to make contact with bone tunnels, and no complications associated with screws.(Buranapuntaruk et al., 2021).

Therefore, we propose a novel graft strategy that utilizes LARS ligament to augment autografts. We think this could potentially combine the beneficial healing properties of autologous tendons with the robust strength of artificial ligaments. In this study all-inside ACL reconstruction technique was adopted. Both sides of the femur and tibia were fixed with Endo-Button and rigidloop titanium plates. Then, the early clinical effects of mixed tendon were compared with those of autologous tendon transplantation alone in ACL reconstruction.

## 2 Patients and methods

### 2.1 Clinical data

Based on previous studies (Ebert and Annear, 2019; Aujla et al., 2021; Zhang et al., 2022), the mean difference in Tegner scores was estimated as 0.8 with a standard deviation (SD) of 1.2, whereas the other scores showed a mean difference of 5 and an SD of 8. Based on these estimates, we used PASS 2021 (version: 21.0.3) with α = 0.05 and power = 0.8 to calculate the sample sizes, which were estimated as 37 and 42 per group, respectively. Considering a 10% attrition rate, each group required at least 46 samples.

All patients aged ≥16 with ACL injuries requiring ACL reconstruction were eligible for evaluation in this study. The exclusion criteria included 1) patients with bilateral ACL injury requiring surgical reconstruction; 2) patients with other knee ligament injuries, such as medial collateral ligament and posterior cruciate ligament, requiring surgical repair; 3) patients failing to comply with the study protocol; 4) patients who cannot understand study information or complete questionnaires due to cognitive or language limitations; and 5) patients with complications at risk of surgery.

From June 2023 to September 2024, 121 patients were initially eligible for evaluation; however, 7 patients did not meet the inclusion criteria, and 15 patients declined to participate. Finally, 99 patients participated in the study. All patients were required to provide written informed consent before participation, ensuring they completely understood the procedures and potential risks. Moreover, all surgical procedures were performed by the same experienced surgeon to maintain consistency and reduce variability in the outcomes. Table 1 shows the baseline data of the included patients. Participants were allocated using a single-blind quasi-randomization method, wherein the allocation· sequence was concealed from the patients to reduce allocation bias. The inclusion and exclusion criteria were approved, and patients were assigned study numbers. In the augmentation group (hamstring tendons augmented using the LARS internal brace ligament), 48 patients were included, of whom 15 underwent meniscectomy, 24 received meniscal repair, and 9 received no meniscal operation. In the hamstring group (hamstring tendons only) consisting of 51 patients, 17 underwent meniscectomy, 26 underwent meniscal repair, and 8 did not undergo meniscal operation. The two groups were similar in terms of gender, age, time from injury to surgery, and preoperative knee function scores.

**TABLE 1:** General characteristics of patients.

Variable	Total (n = 99)	Augmentation (n = 48)	Hamstring (n = 51)	P Value
Mean age (year)	29.86 ± 10.61	29.1 ± 9.46	30.71 ± 11.76	0.442
Gender				
Male	70 (70.7)	38 (79.2)	32 (62.7)	0.116
Female	29 (29.3)	10 (20.8)	19 (37.3)	
Knee				0.265
Right	48 (48.5)	20 (41.7)	28 (54.9)	
Left	51 (51.5)	28 (58.3)	23 (45.1)	
Mechanism of injury				0.349
Sports	74 (74.7)	36 (70.6)	38 (79.2)	
Traffic accidents	18 (18.2)	12 (23.5)	6 (12.5)	
Other	7 (7.1)	3 (5.9)	4 (8.3)	
Time from injury to surgery (months)	1.85 ± 0.52	1.81 ± 0.45	1.89 ± 0.58	0.461
BMI (kg/m^2^)	23.25 ± 2.68	23.04 (2.33)	23.44 ± 2.99	0.462
Meniscus situations				
Resection	32 (32.3)	15 (31.2)	17 (33.3)	0.917
Repair	50 (50.5)	24 (50.0)	26 (51.0)	
Conservative	17 (17.2)	9 (18.8)	8 (15.7)	

Augmentation, LARS augmentation; Hamstring, hamstring autograft.

Values are expressed as mean ± SD or no. (%) of patients.

### 2.2 Operation method

#### 2.2.1 Preparation of Graft for the augmentation group

**FIGURE 1:**
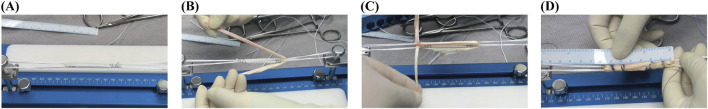
Preparation of Graft for the Augmentation Group. Notes: **(A)** LARS ligament core; **(B)** and **(C)** Autologous tendon wrap; **(D)** Integrated by weaving and sewing.

The hamstring tendon of the affected limb was excised using a standard surgical method. A single strand of appropriately sized hamstring tendon was combined and woven with the LARS internal brace ligament and then folded into double strands, with the synthetic material completely enveloped within the hamstring tendon. High-strength sutures were used to secure both ends, completing the preparation of the graft, which had a diameter of 8 mm. The hamstring group was prepared using the hamstring tendon only (Figure 1).

#### 2.2.2 Arthroscopic Graft Installation

**FIGURE 2:**
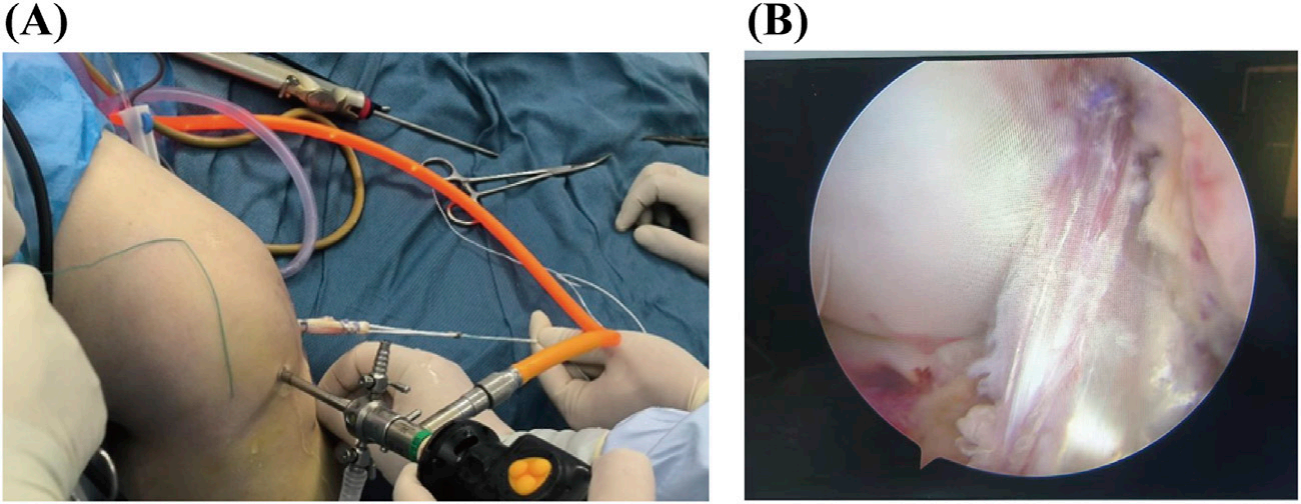
Arthroscopic Graft Installation. **(A)** Pull the tendon in using a full-thickness reconstruction method; **(B)** The reconstructed tendon.

A standard anteromedial and anterolateral approach was used for the arthroscopic examination. Efforts were made to preserve any remaining ACL and synovial tissue as much as possible. All patients underwent arthroscopic single-bundle ACL reconstruction. To align with the footprint of the anteromedial bundle, an appropriate femoral offset guide was positioned at either the 1 o’clock or 11 o’clock orientation, depending on whether the procedure involved the left or right knee. The tibial tunnel was carefully placed as far anteriorly as possible to minimize potential interference with the intercondylar fossa. Fixation of the graft at both the femoral and tibial ends was achieved using Endo-Buttons, which were secured with titanium plates and straps (Figure 2).

### 2.3 Postoperative rehabilitation

The patient was released on the day after surgery, and the standardized rehabilitation protocol was followed (Kotsifaki et al., 2023). Quadriceps isometric contractions and ankle pumps were performed immediately after surgery, and the wound was iced. Patients who did not undergo meniscus repair were allowed to return to full weight-bearing within 2 weeks. For patients who underwent meniscal repair simultaneously, the rehabilitation period was typically extended to 4 weeks. Swimming and cycling activities are typically permitted after 6 weeks of recovery, provided that there are no complications or concerns with the healing process. Straight running and jogging can generally be resumed around the 3-month follow-up, as long as joint stability and muscle strength have sufficiently improved. More complex and dynamic movements, such as cutting and spinning, were introduced cautiously after 6 months to minimize the risk of reinjury and ensure that the graft has completely integrated and healed. Regular follow-up and professional evaluation are crucial throughout this timeline to adjust the rehabilitation plan based on individual progress. Specific rehabilitation measures were guided and adjusted by professional surgeons according to the patients’ recovery status.

At 3 months postoperatively, Lachman and pivot shift tests were performed to evaluate knee joint stability. All physical examinations and clinical evaluations were meticulously conducted by a skilled and experienced technician who was not involved in any aspect of patients’ rehabilitation treatment. This separation was implemented to eliminate potential bias and ensure objective and reliable evaluation results. Patient scores were collected during routine follow-ups before surgery and at 1, 3, and 6 months postoperatively. Subjective outcomes consisted of the Lysholm and Tegner activity scale scores, and the ACL-RSI (anterior cruciate ligament-return to sport after injury) scale was used to evaluate the psychological degree for resuming sports after ACL reconstruction.

The KOS-ADLS (Knee Outcome Survey–Activities of Daily Living Scale) was used to quantify the degree of disability in performing daily living tasks. Furthermore, the IKDC (International Knee Documentation Committee) form was used to evaluate the current functional capacity. MRI and CT scans were performed when necessary.

### 2.4 Tensile test

The specific protocol for the tensile test was performed as described in previous studies (Barber et al., 2019; Kamada et al., 2022; Kanayama et al., 2023). Considering that bovine tendons and human hamstring tendons have similar strength, we used bovine tendons as a substitute for human tendons in this experiment (Domnick et al., 2016; Barber et al., 2019). We used an INSTRON universal testing machine to test the three types of grafts (each type was tested three times as follows: A: tendons group, using bovine tendons; B: tendons + LARS group, using bovine tendons augmented with LARS internal brace ligament; C: LARS group, using LARS internal brace ligament only). The distal end of the graft was clamped using fixtures connected to the mechanical testing machine. The traction test was conducted at a speed of 5 mm/min until the ligament failed or reached the maximum capacity of the testing machine, generating displacement–load curves (A, B, and C).

### 2.5 Establishment of the ACLR model

**FIGURE 3:**
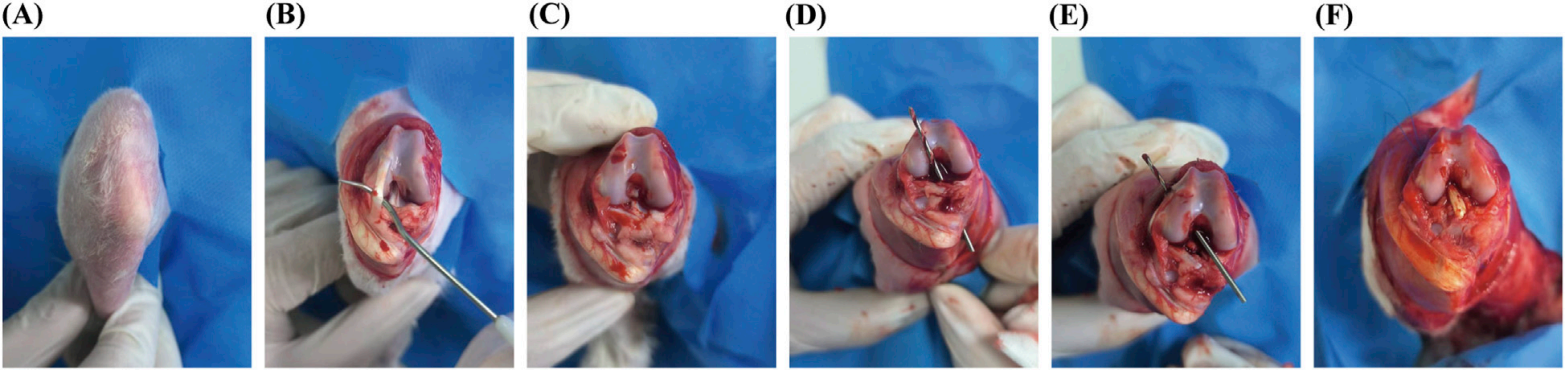
Establishment of the ACLR model. **(A)** Preparation and disinfection of the surgical site; **(B)** Incise the knee joint and locate the extensor digitorum longus; **(C)** Harvest the EDL tendon and transect the anterior cruciate ligament; **(D)** and **(E)** Create a tibial–femoral tunnel; **(F)** Pull the graft into the bone tunnel.

To examine the postoperative healing efficacy of augmentation ligaments, we used 12 3-kg male New Zealand white rabbits to establish an ACL model, according to a methodology similar to previously described procedures (Liu et al., 2023;; Leite et al., 2024). In each rabbit, an incision was made at the medial malleolus and the plantar part of the foot to excise the extensor digitorum longus (EDL) tendon for graft preparation (Figure 3). The graft consisted of a hybrid combination of a synthetic LARS ligament and the rabbit’s autologous EDL tendon. Both ends of all grafts were secured using 4–0 sutures. A medial approach was used to access the knee joint of the ipsilateral limb, and tunnels were created through the tibia and femur using a 2-mm drill. The graft was then passed through these tunnels, with its ends anchored to the periosteum surrounding the cortical bone and at the tunnel exits. Postoperatively, the animals were allowed to move freely. At 4 and 8 weeks postsurgery, animals from different groups were euthanized by administering an anesthetic overdose.

### 2.6 Histological analysis

After decalcification, the samples were embedded in paraffin and sliced perpendicularly to the tibial and femoral tunnels, with a thickness of 5 μm. Hematoxylin and eosin (H&E) and Masson’s trichrome staining were used to examine the formation of new bone and collagen fibers at the tendon–bone junction, as well as the infiltration of cells within the artificial LARS ligament and the formation of collagen fibers.

### 2.7 Statistical analysis

Patient data were analyzed using GraphPad Prism (version: 9.5.0) and SPSS (version: 25). If data followed a normal distribution, ANOVA was applied; otherwise, the Mann–Whitney U test was used. P < 0.05 was considered statistically significant. Continuous variables are expressed as mean ± SD, and categorical variables are presented as percentages.

## 3 Results

### 3.1 Knee joint function score

**FIGURE 4:**
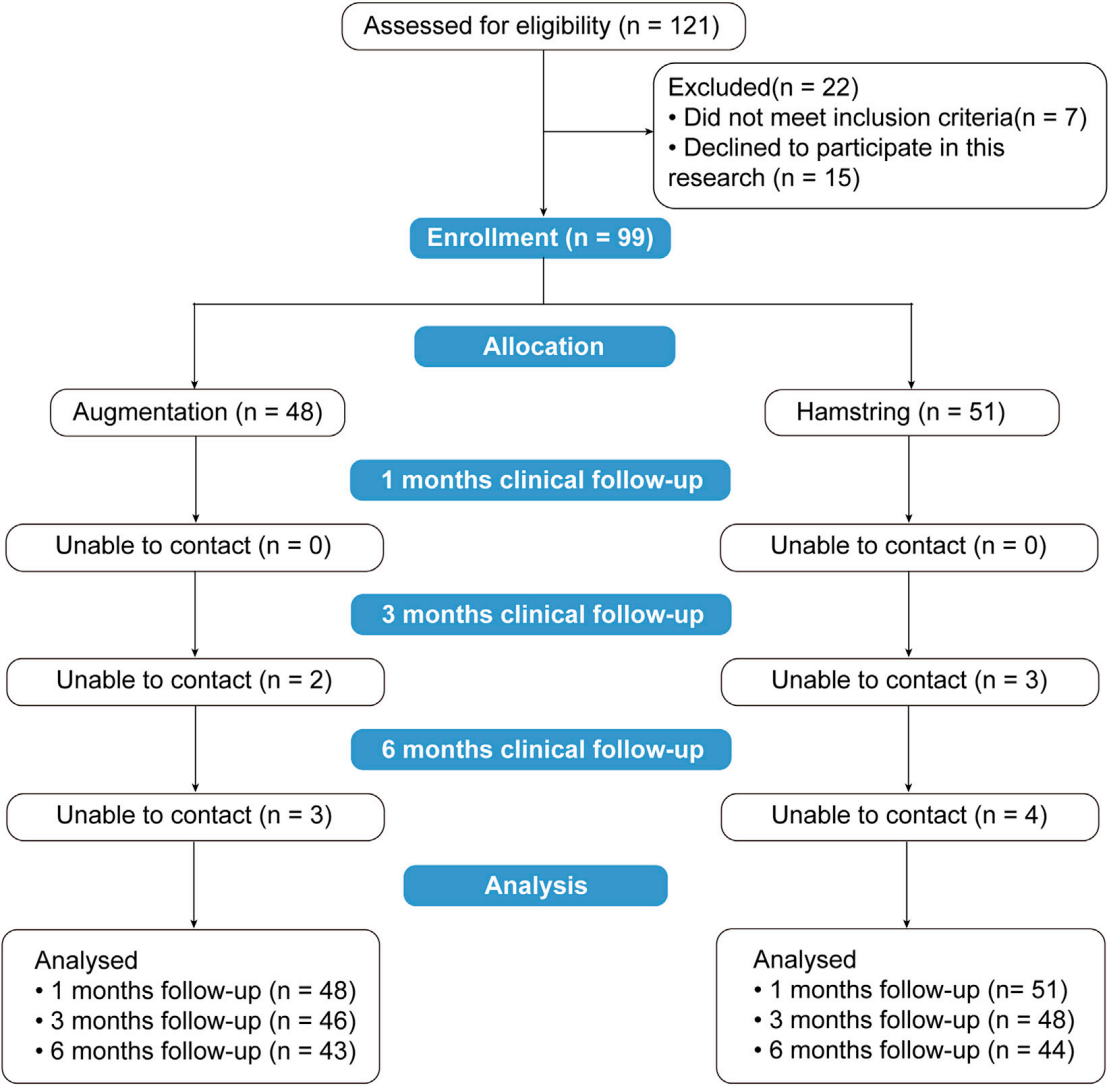
Flow diagram.

A total of 121 patients underwent ACL reconstruction between June 2023 and March 2024. After excluding 22 patients, 99 were included, of whom 48 received tendon augmented with LARS internal brace ligament and 51 received hamstring tendon autograft. After 3 months, 5 patients were lost to follow-up, and by 6 months, an additional 7 patients were lost, resulting in 87 patients remaining. A total of 12 patients were lost to follow-up within 6 months (Figure 4).

As shown in Table 2, there were significant differences between the two groups in the Lysholm and KOS-ADLS scores at the 1- and 3-month time points, with the augmentation group exhibiting higher scores than the hamstring group. However, the ACL-RSI scores were not significantly different between the hamstring and augmentation groups at the 1-, 3-, and 6-month postoperative time points. Furthermore, remarkable discrepancies were observed in the Tegner scores at the 3- and 6-month time points, as well as in the IKDC score at the 1-month follow-up, all of which favored the augmentation group in terms of early postoperative outcomes compared with the hamstring group.

**TABLE 2:** Comparison of patient-reported outcomes between the two groups.

Follow-up time	No. of patients		ACL-RSI		Tegner scores		Lysholm scores		KOS-ADLS scores		IKDC scores	
	Hamstring	Augmentation	Hamstring	Augmentation	Hamstring	Augmentation	Hamstring	Augmentation	Hamstring	Augmentation	Hamstring	Augmentation
Preoperative	51	48	58.8 ± 22.0	54.8 ± 21.5	1.6 ± 1.1	1.5 ± 1.0	52.7 ± 5.1	51.2 ± 5.6	53.2 ± 8.5	50.5 ± 6.4	51.4 ± 3.8	49.9 ± 4.2
1 month	51	48	61.9 ± 20.2	63.2 ± 20.0	1.7 ± 1.1	2.0 ± 1.1	61.7 ± 7.0	65.3 ± 6.3*	61.1 ± 9.8	66.4 ± 10.7*	64.7 ± 6.9	68.8 ± 5.8**
3 months	48	46	66.3 ± 18.2	71.8 ± 16.7	2.5 ± 1.3	3.3 ± 1.2**	71.9 ± 6.4	75.7 ± 4.6**	72.7 ± 8.2	77.5 ± 7.9*	75.7 ± 7.8	76.4 ± 7.1
6 months	44	43	72.4 ± 17.3	75.9 ± 15.6	3.4 ± 1.1	4.1 ± 1.3*	82.9 ± 5.9	84.7 ± 6.4	82.6 ± 8.9	85.6 ± 9.2	83.0 ± 6.4	83.6 ± 6.0

Augmentation, LARS augmentation; Hamstring, hamstring autograft.

Values are expressed as mean ± SD.

*Indicates P < 0:05, **represents P < 0:01, ***signifies P < 0:001.

We performed subgroup analyses to investigate the effectiveness of augmented ligaments in diverse patient populations. As shown in Supplementary Table S1, for individuals aged 16 to 35, the augmentation group demonstrated superior scores compared to the hamstring group in the Tegner score at the 1- and 3-month follow-up, the Lysholm score at the 3-month follow-up, and the KOS-ADLS score at the 1-month follow-up. In the age range of 36–56, notable disparities emerged between the groups in Lysholm (at 3 month) and IKDC (at 1 month) scores again favoring the augmentation group. Among males, significant variations were solely noted in Lysholm scores at the 3-month follow-up. For females, significant differences between the groups were evident in Lysholm, KOS-ADLS, and IKDC scores at 1 month, as well as in Tegner and KOS-ADLS scores at 3-months. This suggested that augmented ligaments may be more suitable for young female populations.

### 3.2 Physical examination results

At 3 months postsurgery, an experienced clinician who was not involved in the experiment conducted Lachman tests, anterior drawer tests, and pivot shift tests on the patients and reported no significant differences between the augmentation and hamstring groups (Table 3).

**TABLE 3:** Lachman, drawer, and pivot shift tests 3 months after surgery.

Group	Pivot shift				Drawer			Lachman		
	—	Ⅰ	II	III	—	±	+	—	±	+
Hamstring (n = 51)	50	1			50	1		50	1	
Augmentation (n = 48)	48				48			48		

### 3.3 Postoperative complications

Among the 51 patients in the hamstring group, 1 patient experienced early wound infection, which resolved after 2 weeks of oral antibiotics, as evidenced by a negative synovial fluid bacterial culture. There were no abnormalities in the augmentation group. To better evaluate complications, reoperations, and graft failures, all patients who underwent surgery during the study period were contacted via phone for follow-up. This was done irrespective of their level of participation in the evaluation. Besides the abovementioned case, there were no incidents of ligament laxity, infection, synovitis, tunnel enlargement, or tears in either group.

### 3.4 MRI results

**FIGURE 5:**
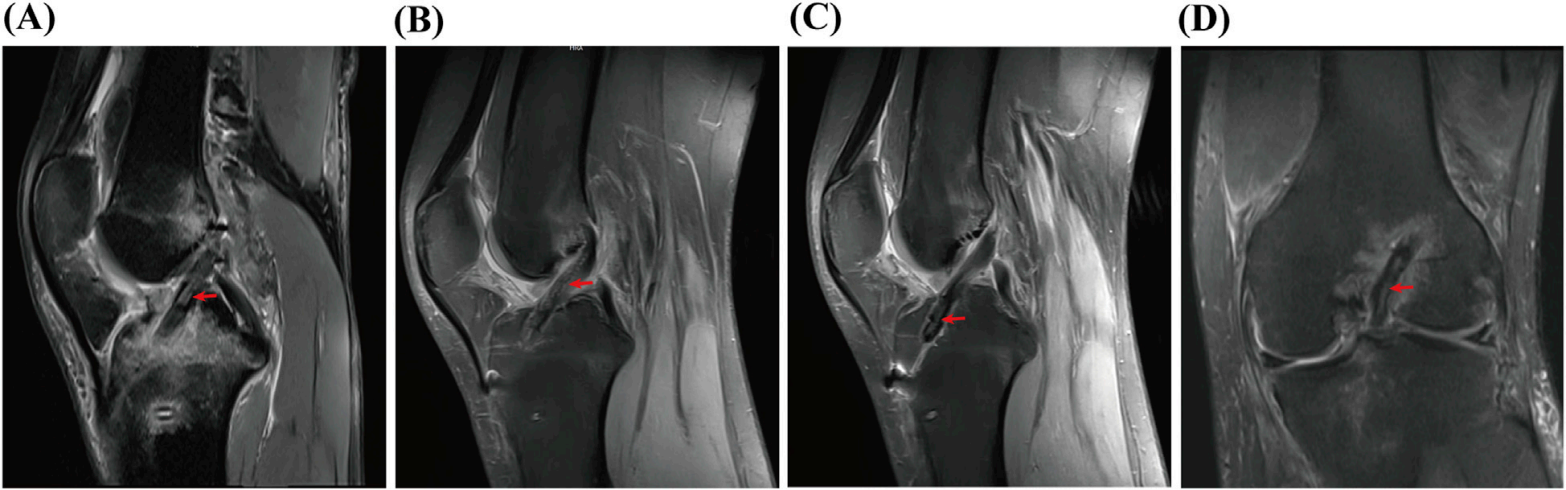
MRI results. **(A-C)** are sagittal MRI images. **(D)** is a coronal MRI image. Red arrow: the position of the augmented ligament.

MRI examinations performed 3 months postsurgery indicated good healing in augmentation group. In the MRI scans of the augmentation group, on the sagittal plane, the graft exhibited normal intraarticular morphology with the autologous tendon wrapped in synthetic material; on the coronal plane, the femoral tunnel was completely filled with the graft (Figure 5).

### 3.5 CT results

**FIGURE 6:**
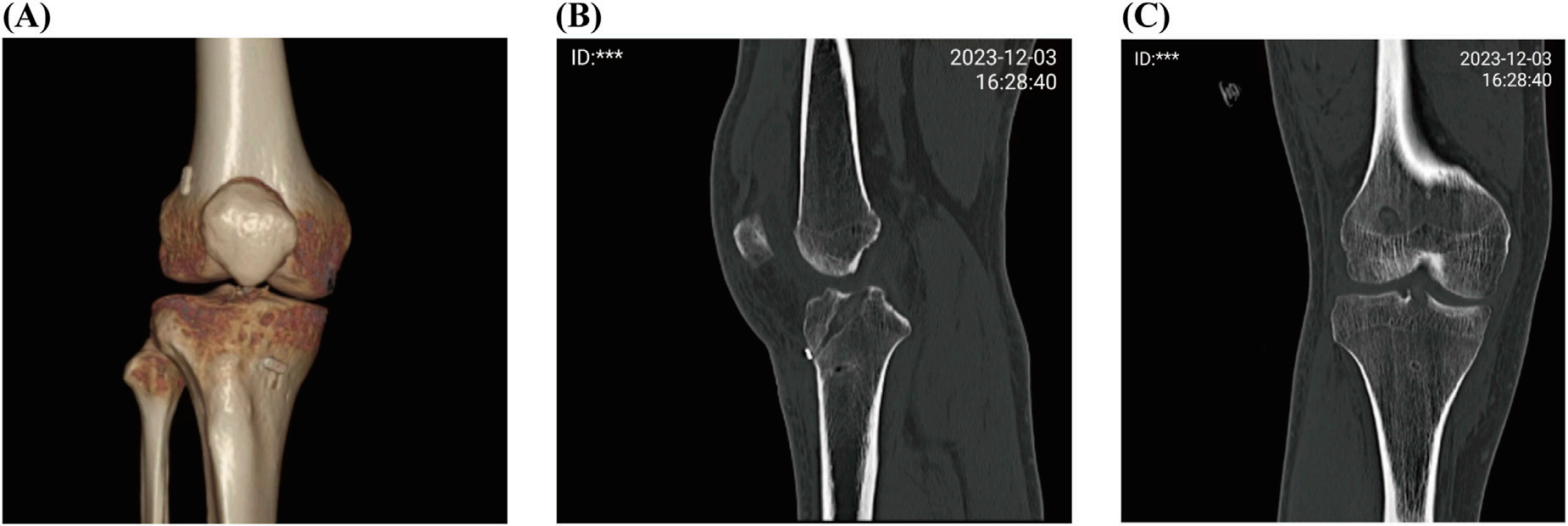
CT results. **(A)** is a three-dimensional reconstructed image. **(B)** is a sagittal CT image. **(C)** is a coronal CT image.

CT images revealed that the titanium plates with straps on the lateral condyle above the distal femur and the medial side of the proximal tibia were closely attached to the bone cortex. The bone tunnel through which the tendon passes was well-shaped and without enlargement (Figure 6).

### 3.6 Tensile test results

**FIGURE 7:**
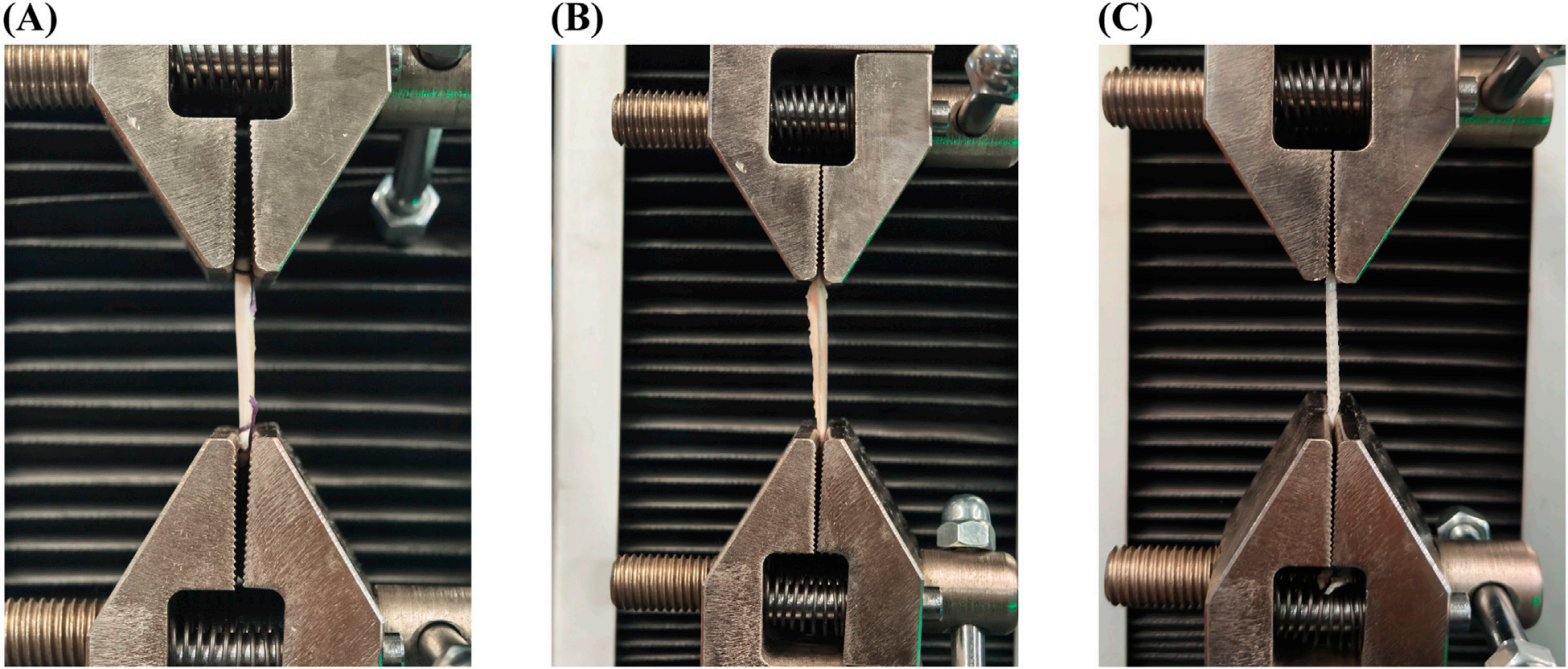
Tensile test machine. **(A)**: tendons group, using bovine tendons; **(B)**: tendons + LARS group, using bovine tendons augmented with LARS internal brace ligament; **(C)**: LARS group, using LARS internal brace ligament only.

**FIGURE 8:**
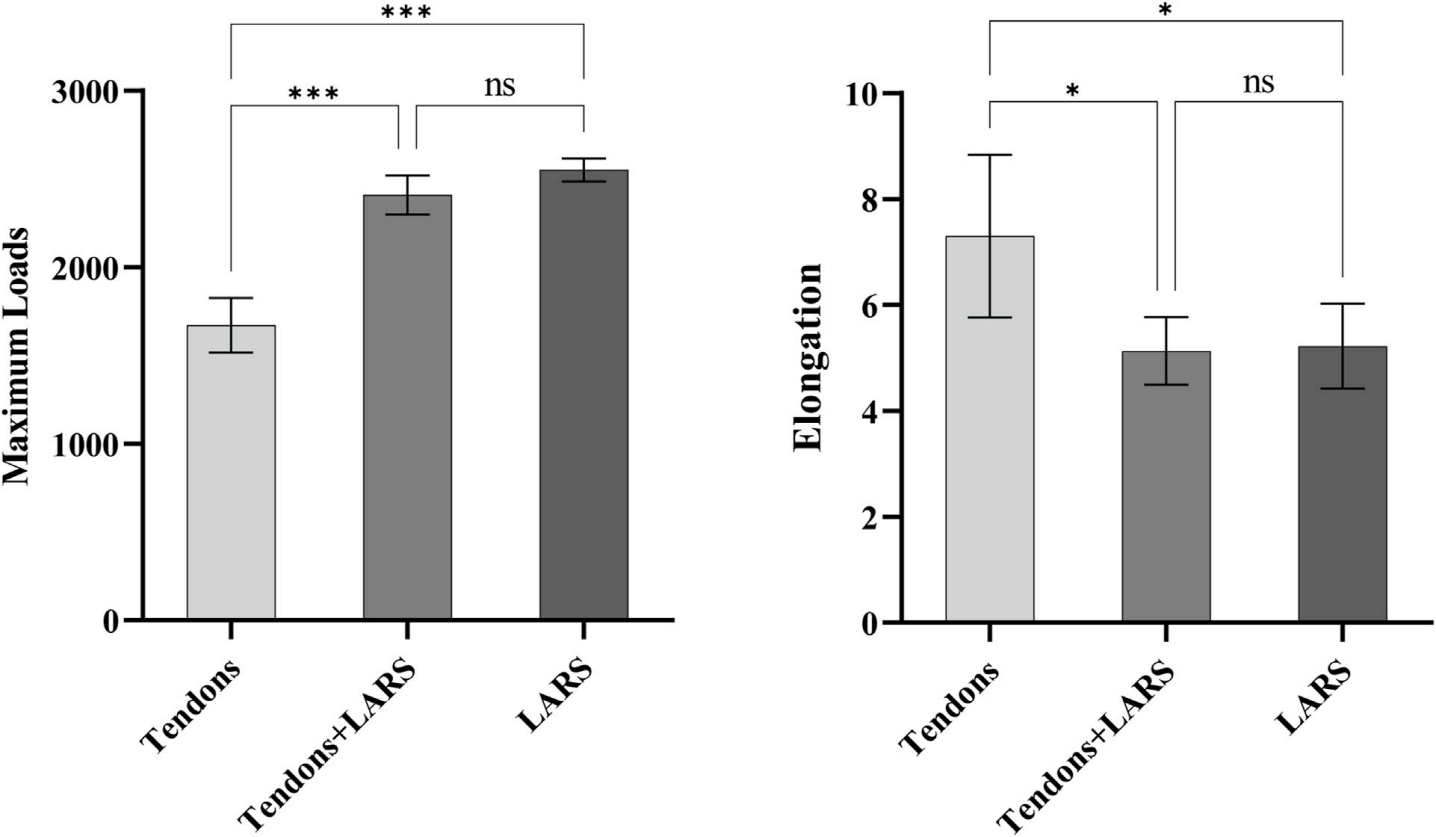
Tensile test.

The ends of ligaments were fixed on the tensile testing machine (Figure 7). The tensile strength testing (Table 4; Figure 8) confirmed that the tendon group exhibited the weakest performance, with significantly lower maximum loads (1671.8 ± 154.62) than the tendons + LARS (2,410 ± 111.36) and LARS (2551.6 ± 64.38) groups (P < 0.001). Moreover, the tendon group exhibited significantly higher elongation (7.30 ± 1.54) than both the tendons + LARS (5.13 ± 0.64) and LARS (5.22 ± 0.80) groups (P < 0.05). There were no significant differences between the tendons + LARS and LARS groups in terms of maximum loads or elongation.

**TABLE 4:** Tensile strength testing of various types of graft materials.

Groups	Maximum load	Elongation
Tendons	1671.8 ± 154.62	7.30 ± 1.54
Tendons + LARS	2,410 ± 111.36	5.13 ± 0.64
LARS	2551.6 ± 64.38	5.216 ± 0.80

Values are expressed as mean ± SD.

### 3.7 Animal experiment results

After dissecting the joints of the rabbits, at both the 4- and 8-week postsurgery intervals, the surface of the reconstructed ACL was found to be completely covered with connective tissue. These findings in the rabbit model suggest that the augmented graft integrates effectively with surrounding tissues, supporting the clinical improvements observed in human patients.

**FIGURE 9:**
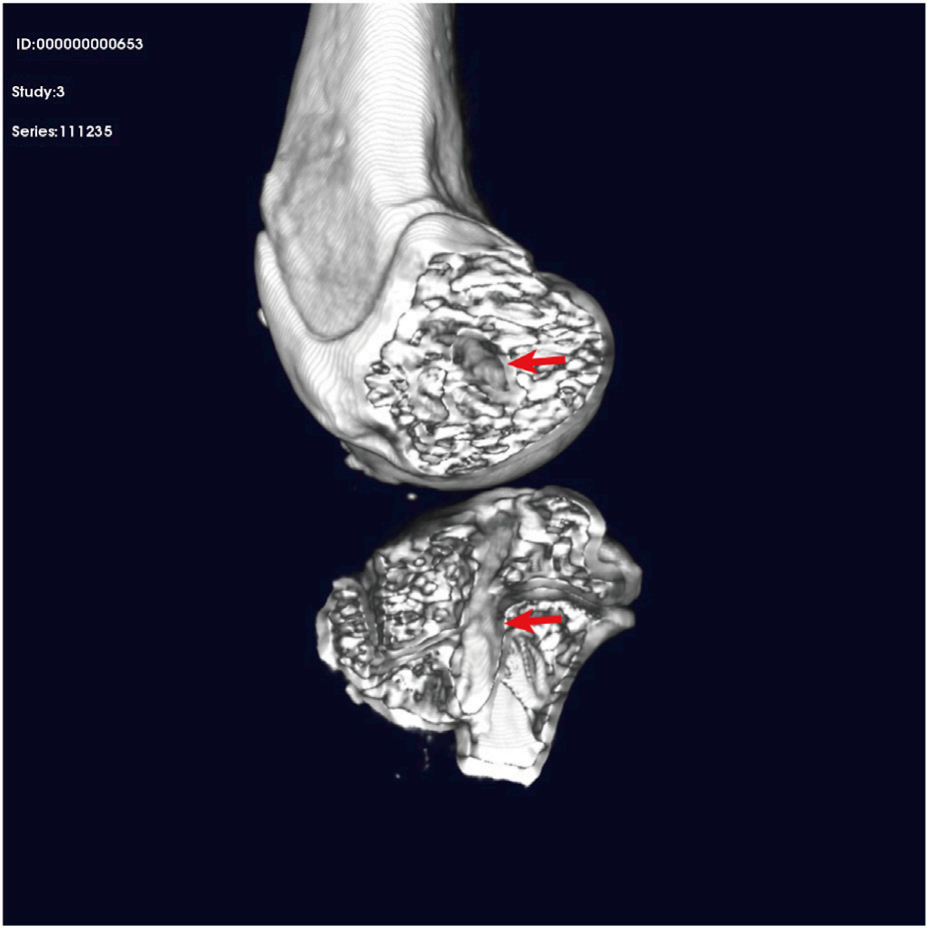
Micro-CT. Red arrow: position of the bone tunnel.

Micro-CT images revealed an intact bony tunnel in the knee joint of the animal with no enlargement of the tibiofemoral bony channel (Figure 9).

**FIGURE 10:**
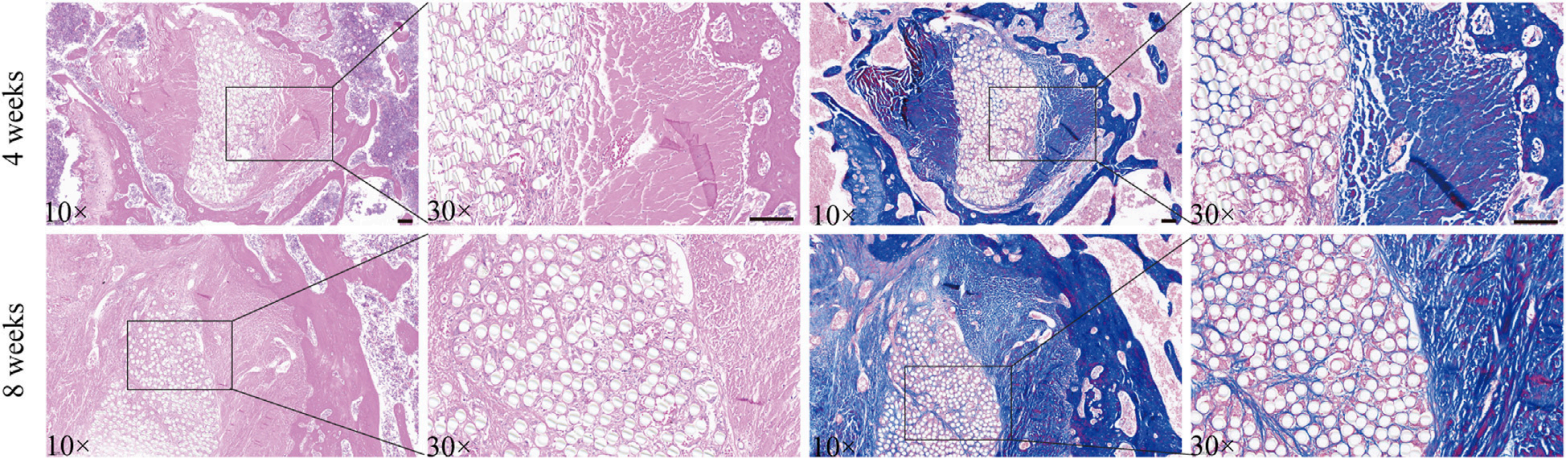
Stained animal tissue sections. Notes: The left side shows H&E staining results, and the right side shows Masson’s trichome staining results. Scale bar = 100 μ m.

At 4 weeks postimplantation, H&E staining revealed that the artificial LARS ligament fibers in the rabbits were encapsulated by fibrous connective tissue, with neovascularization evident within this tissue (Figure 10). However, there was a gap between the artificial material and collagen fibers. The autologous EDL tendon exhibited fibrous connections with the bone tissue, although these connections were not robust. By 8 weeks, there was a marked increase in the infiltration of fibrous connective tissue surrounding the artificial LARS ligament fibers compared with that at 4 weeks; this reduction in the gap between the artificial material and collagen fibers in the animal model could indicate improved graft integration, which may result in better long-term stability and reduced failure rates in clinical applications. Moreover, the fibrous tissue connections between the autologous EDL tendon and bone tissue became more compact. Masson’s trichrome staining revealed minimal collagen between the artificial LARS ligament fibers in the rabbits at 4 weeks postimplantation. By 8 weeks, there was a remarkable increase in the formation of collagen fibers.

## 4 Discussion

This study investigated the early clinical outcomes of different grafts in ACL reconstruction. The most important finding of our study was that the autologous tendon mixed with the LARS intraligamentous enhancement technique yielded higher clinical scores and patient satisfaction early after ACL reconstruction, as well as good mechanical properties and tissue growth characteristics. Furthermore, none of the patients had any postoperative complications. These results support our hypothesis that the combination of autologous tendons and LARS artificial ligaments improves clinical outcomes by providing superior mechanical strength and promoting tissue integration. Four hamstring tendons and bone-patellar tendons are the most frequently used autografts and were once considered to remain the standard for ACL reconstruction. (Kim et al., 2013; Budny et al., 2017). This study provides a novel option for patients with thin autografts.

The concept of an artificial replacement for ACL was first proposed in 1918 by Smith, who used a graft secured in place with staples and multiple silk sutures. Unfortunately, the surgery was unsuccessful, and the graft failed shortly after implantation. Since the 1970s, the use of artificial ligaments has increased due to the advancement of novel materials and technologies, including Teflon, carbon fiber, Gore-Tex, polyester, and later the Leeds-Keio and Kennedy-Ligament strengthening devices (LAD). Nevertheless, due to their various limitations, all were abandoned (Bolton and Bruchman, 1985; Fujikawa et al., 1989; Richmond et al., 1992; Rading and Peterson, 1995).

LARS represents a new generation of synthetic ligaments, featuring remarkable advancements in overall design, weaving technology, and surgical techniques compared with previous synthetic ligaments (Chinese Specialist Consensus Group On New Generation Artificial Ligaments Used For Anterior Cruciate Ligament, 2022). Unlike other biological grafts, LARS is made from PET fibers and does not undergo the “necrosis-vascularization–recombination” tissue regeneration process (Chen and Chen, 2020). LARS depends on “isometric” or “near-isometric” techniques in ACL reconstruction, requiring precise bone tunnel positioning to compensate for its poor elasticity. In addition, the tissue growth characteristics of LARS ligaments within bone tunnels have been questioned. In the present study, the LARS ligaments were completely encased in autologous tendons, and the two demonstrated good biological integration (Viateau et al., 2013).

Historically, the improvement of ACL grafts, often referred to as “internal brace”, is not a new concept, with their introduction dating back to 1980. (Kennedy et al., 1980). Sutured band augmentation and LARS ligament augmentation are common. Biomechanically, ACL graft augmentation can protect the graft from irreversible elongation and improve the biological fusion of the graft, especially during the maturation and remodeling stages of healing (Noonan et al., 2020; Millan et al., 2021; Smith, 2021).

The advantage of LARS intraligament enhancement lies in the combination of LARS ligaments with autologous tendons, which ensures the thickness and length of the graft. This is especially beneficial for patients with naturally thin autologous tendons. According to (Snaebjornsson et al., 2017), for every 0.5 mm increase in the diameter of a hamstring autograft, the probability of revision surgery decreased by 0.85 times.

In this study, we employed the all-inside ACL reconstruction approach. Compared to the traditional full-length bone tunnel method, the all-inside technique creates bone tunnels in the femur and tibia that are wider internally and narrower externally, preserving more bone mass and cortical bone integrity, and reducing the risk of postoperative complications such as fractures (Kocabey et al., 2019; Kouloumentas et al., 2019). Additionally, the all-inside technique avoids the need for interference screw fixation at the tibial end, thereby reducing the required graft length. When combined with internal augmentation techniques, it not only decreases the length requirement but also achieves an ideal graft diameter, making it particularly suitable for patients with multiple ligament injuries or insufficient autologous ligaments. Moreover, through all-inside reconstruction, the LARS internal brace ligament provides early strength support, enabling early patient mobilization. Simultaneously, the hamstring tendon wrapped around its periphery is more likely to heal with the bone tunnel, thereby promoting patient recovery. This approach aligns with the current concept of ERAS (Enhanced Recovery After Surgery)

In animal experiments, a mixed internally enhanced graft composed of the rabbit EDL tendon and LARS ligament was implanted into the rabbit knee joint. Micro-CT images showed that the animal’s bony tunnel was intact in shape and not enlarged. Histologically, hybrid grafts have a better prognosis than LARS ligaments alone. H&E and Masson’s trichrome staining revealed cellular infiltration between the LARS ligament fibers and the generation of collagen fibers and neovascularization.

Regarding the cost-effectiveness of the new technique, although the use of LARS ligament augmentation may incur additional medical expenses, these costs are generally covered by insurance, thereby limiting the financial burden on patients. Furthermore, the application of internal brace in reconstruction accelerates patient recovery, reducing the need for additional caregiving and rehabilitation expenses. In this study, the augmented ligaments demonstrated excellent performance, with no cases of failure and few postoperative complications, which reduced the costs associated with retreatment and revision surgeries.

In summary, we believe this graft offers several advantages: 1. The outer autografts directly contacts the bone tunnel, which is more conducive to tendon-bone healing compared to LARS ligaments and allografts. 2. The inner LARS ligament provides support during the early stages of reconstruction, reducing the risk of early failure of the autografts. 3. The filling effect of the inner LARS ligament allows for achieving a graft diameter of 8 mm with less tendon harvested. It not only minimizing donor site morbidity but also enabling the use of autografts reconstruction in lean individuals.

This study has several advantages. First, it presents a novel method for ligament weaving. Second, it demonstrates the feasibility of reinforcing ligaments from clinical, biomechanical, and histological perspectives, making the conclusions more reliable. Nevertheless, there are also several limitations in this study. First, the patient was discharged from the hospital the day after the surgery. Second, although rehabilitation was conducted under the supervision of professional surgeons, due to individual differences and varying levels of compliance, controlling the rehabilitation process was challenging, which may have affected postoperative functional recovery. Finally, the sample size and follow-up period were limited. Therefore, continuous and close follow-up is required to evaluate the long-term effects on patient prognosis and reinjury rates.

## 5 Conclusion

Using the LARS ligament augmentation technique for ACL reconstruction yields superior clinical outcomes, biomechanical properties, and histological results than traditional methods. It provides a novel solution for patients with ACL injuries who have thin autologous tendons.

## Data Availability

The raw data supporting the conclusions of this article will be made available by the authors, without undue reservation.

## References

[B2] Ajrawat P. Dwyer T. Whelan D. Theodoropoulos J. Murnaghan L. Bhargava M. (2021). A comparison of quadriceps tendon autograft with bone-patellar tendon-bone autograft and hamstring tendon autograft for primary anterior cruciate ligament reconstruction: a systematic review and quantitative synthesis. Clin. J. Sport Med. 31 (4), 392–399. 10.1097/jsm.0000000000000765 31233432

[B4] Aujla R. S. Ebert J. R. Annear P. T. (2021). Anterior cruciate ligament reconstruction using autologous hamstrings augmented with the ligament augmentation and reconstruction system versus hamstrings alone: a comparative cohort study. Orthop. J. Sports Med. 9 (10), 23259671211046631. 10.1177/23259671211046631 34708141 PMC8543570

[B5] Barber F. A. Howard M. S. Piccirillo J. Spenciner D. B. (2019). A biomechanical comparison of six suture configurations for soft tissue-based graft traction and fixation. Arthroscopy 35 (4), 1163–1169. 10.1016/j.arthro.2018.10.140 30871909

[B6] Beard D. J. Davies L. Cook J. A. Stokes J. Leal J. Fletcher H. (2022). Rehabilitation versus surgical reconstruction for non-acute anterior cruciate ligament injury (ACL SNNAP): a pragmatic randomised controlled trial. Lancet 400 (10352), 605–615. 10.1016/s0140-6736(22)01424-6 35988569

[B8] Bolton C. W. Bruchman W. C. (1985). The GORE-TEX expanded polytetrafluoroethylene prosthetic ligament. An *in vitro* and *in vivo* evaluation. Clin. Orthop. Relat. Res. 196, 202–213. 10.1097/00003086-198506000-00027 3888468

[B9] Budny J. Fox J. Rauh M. Fineberg M. (2017). Emerging trends in anterior cruciate ligament reconstruction. J. Knee Surg. 30 (1), 63–69. 10.1055/s-0036-1579788 27018510

[B50] Buranapuntaruk T. Kongrukgreatiyos K. Itthipanichpong T. (2021). All-Inside arthroscopic Anterior cruciate ligament reconstruction and internal brace with recycling suture. Arthrosc. Tech. 10 (11), e2429–e2434. 10.1016/j.eats.2021.07.022 34868844 PMC8626616

[B10] Cai J. Zhang Q. Chen J. Jiang J. Mo X. He C. (2021). Electrodeposition of calcium phosphate onto polyethylene terephthalate artificial ligament enhances graft-bone integration after anterior cruciate ligament reconstruction. Bioact. Mater 6 (3), 783–793. 10.1016/j.bioactmat.2020.08.037 33024899 PMC7527997

[B11] Chen T. Bai X. Bai L. Chan W. S. Chen S. Chen C. (2024). Diagnosis and treatment of anterior cruciate ligament injuries: consensus of Chinese experts part II: graft selection and clinical outcome evaluation. J. Orthop. Transl. 48, 163–175. 10.1016/j.jot.2024.07.002 PMC11385786 39257437

[B12] Chen T. Chen S. (2020). Artificial ligaments applied in anterior cruciate ligament repair and reconstruction: current products and experience. Zhongguo Xiu Fu Chong Jian Wai Ke Za Zhi 34 (1), 1–9. 10.7507/1002-1892.201908084 31939226 PMC8171823

[B13] Chinese Specialist Consensus Group On New Generation Artificial Ligaments Used For Anterior Cruciate Ligament, R (2022). Core techniques and adverse events in anterior cruciate ligament reconstruction using a new generation of artificial ligaments: the consensus of Chinese specialists based on a modified Delphi method (Part 2). Zhongguo Xiu Fu Chong Jian Wai Ke Za Zhi 36 (9), 1047–1055. 10.7507/1002-1892.202206026 36111464 PMC9626301

[B14] Cohen S. B. Sekiya J. K. (2007). Allograft safety in anterior cruciate ligament reconstruction. Clin. Sports Med. 26 (4), 597–605. 10.1016/j.csm.2007.06.003 17920955

[B17] Domnick C. Wieskötter B. Raschke M. J. Schulze M. Kronenberg D. Wefelmeier M. (2016). Evaluation of biomechanical properties: are porcine flexor tendons and bovine extensor tendons eligible surrogates for human tendons in *in vitro* studies? Arch. Orthop. Trauma Surg. 136 (10), 1465–1471. 10.1007/s00402-016-2529-2 27475640

[B18] Ebert J. R. Annear P. T. (2019). ACL reconstruction using autologous hamstrings augmented with the ligament augmentation and reconstruction system provides good clinical scores, high levels of satisfaction and return to sport, and a low retear rate at 2 years. Orthop. J. Sports Med. 7 (10), 2325967119879079. 10.1177/2325967119879079 31696135 PMC6822193

[B19] Fujikawa K. Iseki F. Seedhom B. B. (1989). Arthroscopy after anterior cruciate reconstruction with the Leeds-Keio ligament. J. Bone Jt. Surg. Br. 71 (4), 566–570. 10.1302/0301-620x.71b4.2768298 2768298

[B20] Gans I. Retzky J. S. Jones L. C. Tanaka M. J. (2018). Epidemiology of recurrent anterior cruciate ligament injuries in national collegiate athletic association sports: the injury surveillance program, 2004-2014. Orthop. J. Sports Med. 6 (6), 2325967118777823. 10.1177/2325967118777823 29977938 PMC6024527

[B25] Kamada K. Nagai K. Nagamune K. Hoshino Y. Nakanishi Y. Araki D. (2022). Direct suturing quadriceps tendon to a continuous loop with a suspensory button provides biomechanically superior fixation in ACL reconstruction. Knee Surg. Sports Traumatol. Arthrosc. 30 (7), 2307–2313. 10.1007/s00167-021-06805-3 34807305

[B26] Kanayama T. Nakase J. Kimura M. Yoshimizu R. Yanatori Y. Ishida Y. (2023). Speed whip ripstop technique during anterior cruciate ligament reconstruction using quadriceps tendon results in higher fixation strength. Knee Surg. Sports Traumatol. Arthrosc. 31 (9), 4068–4075. 10.1007/s00167-023-07482-0 37318561

[B29] Kennedy J. C. Roth J. H. Mendenhall H. V. Sanford J. B. (1980). Presidential address. Intraarticular replacement in the anterior cruciate ligament-deficient knee. Am. J. Sports Med. 8 (1), 1–8. 10.1177/036354658000800101 7356793

[B30] Kim H. S. Seon J. K. Jo A. R. (2013). Current trends in anterior cruciate ligament reconstruction. Knee Surg. Relat. Res. 25 (4), 165–173. 10.5792/ksrr.2013.25.4.165 24368993 PMC3867608

[B31] Kocabey Y. Yalçln S. Erdil M. Polat G. (2019). An Alternative Femoral Fixation in All‐Inside Anterior Cruciate Ligament Reconstruction: A Solution for Preventing Possible Graft Loosening. Arthrosc. Tech. 8 (8), e861–e865. 10.1016/j.eats.2019.03.028 31696047 PMC6823732

[B53] Kotsifaki R. Korakakis V. King E. Barbosa O. Maree D. Pantouveris M. (2023). Aspetar clinical practice guideline on rehabilitation after anterior cruciate ligament reconstruction. Br. J. Sports Med. 57 (9), 500–514. 10.1136/bjsports-2022-106158 36731908 PMC11785408

[B32] Kouloumentas P. Kavroudakis E. Charalampidis E. Kavroudakis D. Triantafyllopoulos G. K. (2019). Superior knee flexor strength at 2 years with all-inside short-graft anterior cruciate ligament reconstruction vs a conventional hamstring technique. Knee. Surg. Sports. Traumatol. Arthrosc. 27 (11), 3592–3598. 10.1007/s00167-019-05456-9 30888448

[B36] Leite C .B. G. Leite M. S Varone B. B. Santos G. B. D. Silva M. D. S. Pereira C. A. M. (2024). Hyperbaric oxygen therapy enhances graft healing and mechanical properties after anterior cruciate ligament reconstruction: An experimental study in rabbits. J. Orthop. Res. 42 (6), 1210–1222. 10.1002/jor.25787 38225877

[B38] Liu Y. Havasy J. Green S. Deng X. H. Chen D. Piacentini A. (2023). Short‐term evaluation of bone‐ACL‐bone complex allograft in ACL reconstruction in a rabbit model. J. Clin. Med. 12 (22). 10.3390/jcm12227057 PMC10671951 38002670

[B33] Migliorini F. Pilone M. Schäfer L. Bertini F. A. Giorgino R. Maffulli N. (2025). Allograft versus autograft ACL reconstruction in skeletally immature patients: a systematic review. Br. Med. Bull. 153 (1), ldae020. 10.1093/bmb/ldae020 39657067

[B34] Millan S. M. Thorn D. Ford E. (2021). A novel approach to augmenting allograft hamstring anterior cruciate ligament reconstructions utilizing a resorbable type I collagen matrix with platelet rich plasma. Case Rep. Orthop. 2021, 1–6. 10.1155/2021/5574676 PMC7972856 33777468

[B35] Min J. H. Yoon H. K. Oh H. C. Youk T. Ha J. W. Park S. H. (2024). Graft choice to decrease the revision rate of anterior cruciate ligament reconstruction: a nationwide retrospective cohort study. Sci. Rep. 14 (1), 20004. 10.1038/s41598-024-71068-0 39198526 PMC11358314

[B37] Noonan B. C. Bachmaier S. Wijdicks C. A. Bedi A. (2020). Independent suture tape reinforcement of tripled smaller-diameter and quadrupled grafts for anterior cruciate ligament reconstruction with tibial screw fixation: a biomechanical full construct model. Arthroscopy 36 (2), 481–489. 10.1016/j.arthro.2019.06.036 31901386

[B39] Paschos N. K. (2022). Editorial commentary: addition of the sartorius tendon to small-diameter hamstring anterior cruciate ligament autografts may improve outcomes in pediatric and revision cases. Arthroscopy 38 (5), 1595–1596. 10.1016/j.arthro.2021.12.003 35501023

[B40] Rading J. Peterson L. (1995). Clinical experience with the Leeds-Keio artificial ligament in anterior cruciate ligament reconstruction. A prospective two-year follow-up study. Am. J. Sports Med. 23 (3), 316–319. 10.1177/036354659502300311 7661259

[B41] Richmond J. C. Manseau C. J. Patz R. McConville O. (1992). Anterior cruciate reconstruction using a Dacron ligament prosthesis. A long-term study. Am. J. Sports Med. 20 (1), 24–28. 10.1177/036354659202000107 1532479

[B42] Runer A. Keeling L. Wagala N. Nugraha H. Özbek E. A. Hughes J. D. (2023). Current trends in graft choice for anterior cruciate ligament reconstruction - part I: anatomy, biomechanics, graft incorporation and fixation. J. Exp. Orthop. 10 (1), 37. 10.1186/s40634-023-00600-4 37005974 PMC10067784

[B43] Smith P. A. (2021). Editorial commentary: repair the anterior cruciate ligament when you can: add suture tape augmentation and dress for success. Arthroscopy 37 (4), 1242–1244. 10.1016/j.arthro.2020.12.218 33812527

[B44] Snaebjornsson T. Hamrin Senorski E. Ayeni O. R. Alentorn-Geli E. Krupic F. Norberg F. (2017). Graft diameter as a predictor for revision anterior cruciate ligament reconstruction and KOOS and EQ-5D values: a cohort study from the Swedish national knee ligament register based on 2240 patients. Am. J. Sports Med. 45 (9), 2092–2097. 10.1177/0363546517704177 28460194

[B46] Tiefenboeck T. M. Thurmaier E. Tiefenboeck M. M. Ostermann R. C. Joestl J. Winnisch M. (2015). Clinical and functional outcome after anterior cruciate ligament reconstruction using the LARS™ system at a minimum follow-up of 10 years. Knee 22 (6), 565–568. 10.1016/j.knee.2015.06.003 26122668

[B47] Tischer T. Andriolo L. Beaufils P. Ahmad S. S. Bait C. Bonomo M. (2023). Management of anterior cruciate ligament revision in adults: the 2022 ESSKA consensus part III-indications for different clinical scenarios using the RAND/UCLA appropriateness method. Knee Surg. Sports Traumatol. Arthrosc. 31 (11), 4662–4672. 10.1007/s00167-023-07401-3 37133742 PMC10598192

[B15] Urbanek O. Moczulska‐Heljak M. Wróbel M. Mioduszewski A. Kołbuk D. (2023). Advanced graft development approaches for ACL reconstruction or regeneration. Biomedicines 11 (2), 1075–1081. 10.3390/biomedicines11020507 36831043 PMC9953332

[B48] Viateau V. Manassero M. Anagnostou F. Guérard S. Mitton D. Migonney V. (2013). Biological and biomechanical evaluation of the ligament advanced reinforcement system (LARS AC) in a sheep model of anterior cruciate ligament replacement: a 3-month and 12-month study. Arthroscopy 29 (6), 1079–1088. 10.1016/j.arthro.2013.02.025 23726110

[B49] Zhang B. Xiang P. Bian S. Wang Y. Wang Y. Ma Y. (2022). Early clinical outcomes of ACL reconstruction using semitendinosus tendon combined with LARS synthetic. Comput. Math. Methods Med. 2022, 1–7. 10.1155/2022/2845114 PMC9553347 36238490

