# Supplementary material for: Early clinical outcomes of all-inside arthroscopic anterior cruciate ligament reconstruction with autograft tendon augmentation using the LARS internal brace ligament
Source: Front Bioeng Biotechnol. 2025 Apr 15;13:1556106. doi: 10.3389/fbioe.2025.1556106 (PMC12037631; doi:10.3389/fbioe.2025.1556106)
Supplement: Supplementary file 1 [file Table1.docx]

Supplementary Material

# Supplementary Tables

**Supplementary table 1.** Subgroup comparison of patient-reported outcomes between two groups.

| **Supplementary table 1.** Subgroup comparison of patient-reported outcomes between two groups. | | | | | | | |  |  |  |  |  |  |  |  |  |  |
| --- | --- | --- | --- | --- | --- | --- | --- | --- | --- | --- | --- | --- | --- | --- | --- | --- | --- |
| Follow-up Time | No. of patients | |  | ACL-RSI | |  | Tegner scores | |  | Lysholm scores | |  | KOS-ADLS | |  | IKDC scores | |
|  | Hamstring | Augmentation |  | Hamstring | Augmentation |  | Hamstring | Augmentation |  | Hamstring | Augmentation |  | Hamstring | Augmentation |  | Hamstring | Augmentation |
| Ages 16 to 35 |  |  |  |  |  |  |  |  |  |  |  |  |  |  |  |  |  |
| Pre-operative | 32 | 37 |  | 54.2 ± 19.5 | 58.9 ± 22.8 |  | 1.4 ± 0.9 | 1.5 ± 1.1 |  | 50.4 ± 5.3 | 52.5 ± 5.0 |  | 50.2 ± 6.4 | 52.9 ± 8.3 |  | 49.7 ± 4.2 | 51.7 ± 4.0 |
| 1 months | 32 | 37 |  | 63.1 ± 18.7 | 61.6 ± 20.9 |  | 2.1 ± 0.9 | 1.6 ± 1.0 |  | 64.3 ± 5.6 | 61.5 ± 7.1 |  | 66.1 ± 9.7 | 60.9 ± 9.9* |  | 67.8 ± 5.6 | 64.7 ± 7.2 |
| 3 months | 31 | 36 |  | 71.8 ± 16.0 | 66.3 ± 18.5 |  | 3.3 ± 1.1 | 2.5 ± 1.3* |  | 75.5 ± 4.5 | 71.9 ± 6.6* |  | 77.3 ± 7.9 | 73.0 ± 8.6 |  | 75.6 ± 6.8 | 75.8 ± 8.1 |
| 6 months | 29 | 32 |  | 76.1 ± 15.0 | 72.4 ± 17.9 |  | 4.1 ± 1.4 | 3.4 ± 1.1* |  | 83.8 ± 6.2 | 82.8 ± 5.9 |  | 83.3 ± 9.4 | 82.8 ± 9.4 |  | 83.1 ± 5.2 | 82.9 ± 6.6 |
| Ages 36 to 56 |  |  |  |  |  |  |  |  |  |  |  |  |  |  |  |  |  |
| Pre-operative | 16 | 14 |  | 56.1 ± 25.8 | 58.7 ± 20.8 |  | 1.6 ± 1.3 | 1.9 ± 1.3 |  | 52.7 ± 6.2 | 53.0 ± 5.6 |  | 51.3 ± 6.6 | 54.0 ± 9.3 |  | 50.3 ± 4.4 | 50.7 ± 3.4 |
| 1 months | 16 | 14 |  | 63.3 ± 22.9 | 62.7 ± 19.0 |  | 2.0 ± 1.3 | 1.8 ± 1.3 |  | 67.2 ± 7.4 | 62.4 ± 6.9 |  | 67.0 ± 13.0 | 61.6 ± 10.0 |  | 70.8 ± 6.1 | 64.8 ± 6.3* |
| 3 months | 15 | 12 |  | 71.8 ± 18.6 | 66.5 ± 18.1 |  | 3.3 ± 1.4 | 2.8 ± 1.3 |  | 76.3 ± 4.9 | 71.9 ± 6.0* |  | 78.0 ± 8.3 | 71.9 ± 7.0 |  | 78.0 ± 7.7 | 75.4 ± 7.2 |
| 6 months | 14 | 12 |  | 75.5 ± 17.4 | 72.3 ± 16.1 |  | 4.0 ± 1.3 | 3.5 ± 1.0 |  | 86.5 ± 6.8 | 83.1 ± 6.0 |  | 86.2 ± 9.1 | 81.9 ± 8.0 |  | 84.5 ± 7.6 | 83.3 ± 6.1 |
| Male |  |  |  |  |  |  |  |  |  |  |  |  |  |  |  |  |  |
| Pre-operative | 38 | 32 |  | 54.4 ± 19.9 | 63.3 ± 21.6 |  | 1.4 ± 1.0 | 1.8 ± 1.1 |  | 51.0 ± 5.2 | 53.6 ± 5.3* |  | 50.3 ± 6.0 | 55.2 ± 8.6** |  | 49.8 ± 4.1 | 52.2 ± 3.7* |
| 1 months | 38 | 32 |  | 62.9 ± 18.9 | 66.0 ± 19.8 |  | 2.0 ± 0.9 | 1.9 ± 1.0 |  | 64.5 ± 5.9 | 62.6 ± 6.6 |  | 67.8 ± 10.7 | 62.4 ± 10.0 |  | 68.6 ± 5.6 | 66.0 ± 6.9 |
| 3 months | 36 | 29 |  | 71.7 ± 16.0 | 69.9 ± 18.2 |  | 3.3 ± 1.1 | 2.9 ± 1.1 |  | 75.6 ± 4.6 | 72.3 ± 6.1* |  | 77.4 ± 7.8 | 74.3 ± 7.8 |  | 76.1 ± 6.7 | 77.3 ± 7.7 |
| 6 months | 33 | 25 |  | 76.0 ± 15.0 | 76.1 ± 17.8 |  | 4.0 ± 1.2 | 3.7 ± 0.9 |  | 84.5 ± 6.3 | 83.4 ± 6.1 |  | 85.2 ± 9.2 | 83.3 ± 8.8 |  | 83.1 ± 5.8 | 84.6 ± 6.4 |
| Female |  |  |  |  |  |  |  |  |  |  |  |  |  |  |  |  |  |
| Pre-operative | 10 | 19 |  | 56.3 ± 28.0 | 51.4 ± 21.3 |  | 1.7 ± 1.2 | 1.2 ± 1.2 |  | 51.7 ± 7.2 | 51.2 ± 4.6 |  | 51.6 ± 8.0 | 49.8 ± 7.3 |  | 50.3 ± 4.8 | 50.1 ± 3.8 |
| 1 months | 10 | 19 |  | 64.1 ± 24.9 | 55.1 ± 19.5 |  | 2.4 ± 1.6 | 1.3 ± 1.2 |  | 68.4 ± 7.3 | 60.2 ± 7.6** |  | 68.6 ± 11.7 | 58.9 ± 9.3* |  | 69.6 ± 7.1 | 62.5 ± 6.5* |
| 3 months | 10 | 19 |  | 72.0 ± 20.0 | 60.8 ± 17.2 |  | 3.4 ± 1.6 | 2.0 ± 1.3* |  | 76.2 ± 4.8 | 71.3 ± 7.0 |  | 77.9 ± 8.7 | 70.2 ± 8.3* |  | 77.5 ± 8.8 | 73.1 ± 7.5 |
| 6 months | 10 | 19 |  | 75.7 ± 18.5 | 67.5 ± 15.8 |  | 4.1 ± 1.7 | 3.1 ± 1.2 |  | 85.5 ± 7.0 | 82.2 ± 5.8 |  | 86.9 ± 9.6 | 81.6 ± 9.2 |  | 85.3 ± 6.9 | 80.9 ± 6.1 |
| Augmentation: LARS augmentation, Hamstring: hamstring autograft | | | | | | | | |  |  |  |  |  |  |  |  |  |
| Values are expressed as mean ± SD | | | | | | | | |  |  |  |  |  |  |  |  |  |
| *indicates P < 0.05, **represents P < 0.01, ***signifies P < 0.001 | | | | | | | | |  |  |  |  |  |  |  |  |  |
